# Insulin Signaling in Alzheimer’s Disease: Association with Brain Insulin Resistance

**DOI:** 10.3390/ijms27031222

**Published:** 2026-01-26

**Authors:** Monika Pliszka, Leszek Szablewski

**Affiliations:** Chair and Department of General Biology and Parasitology, Medical University of Warsaw, Chałubińskiego 5, 02-004 Warsaw, Poland; monika.pliszka@wum.edu.pl

**Keywords:** insulin resistance, insulin signaling pathways, PI3K/AKT signaling pathway, MAPK signaling pathway, Alzheimer’s disease

## Abstract

Insulin is an anabolic hormone involved in the regulation of several processes, such as the storage of glucose into glycogen, decrease of glucose output, stimulation of glucose transport into cells, etc. The hormone binds to its receptor, thereby activating an intracellular signaling cascade. Once activated, the insulin receptor (INSR) phosphorylates multiple intracellular substrates, which initiate the downstream signaling pathway. The nature of insulin signaling pathways may vary depending on the organ or tissue. In the central nervous system (CNS), INSRs are expressed in all cell types. This observation may suggest that insulin signaling is involved in important and diverse processes. It regulates glucose metabolism, supports cognitive functions, enhances the outgrowth of neurons, as well as plays a role in the modulation of release and uptake of catecholamine, among other roles. Importantly, insulin can freely cross the blood–brain barrier (BBB) from the circulation and is also synthesized locally within the brain. Insulin resistance (IR) impairs insulin signaling, which may accelerate brain aging, affect plasticity, and potentially contribute to neurodegeneration. Dysregulation of insulin signaling has been implicated in several diseases, including diabetes mellitus, metabolic syndrome, certain cancers, and neurodegenerative diseases, such as Alzheimer’s disease. There are two principal insulin signaling pathways: the PI3K/AKT pathway, primarily associated with metabolic effects, and the MAPK pathway, which is involved in cell growth, survival, and gene expression. Our review describes the role of insulin in the human brain, as well as the disturbances in insulin signaling resulting from brain insulin resistance, with a particular focus on its association with Alzheimer’s disease.

## 1. Introduction

There are several definitions of IR. Most often, it is described as a decreased responsiveness of target cells to physiological levels of insulin. An observed effect of IR is a prolonged decrease in elevated blood glucose levels due to a particular concentration of insulin. Thus, IR causes the lowering of blood glucose to physiological levels, requiring increased amounts of insulin. This condition, known as hyperinsulinemia, develops in parallel with the onset of IR. Insulin is an anabolic hormone, mainly secreted by β-cells in the islets of Langerhans in the pancreas. However, the physiological function of insulin is usually seen in the process of glucose homeostasis, when it exerts a broad range of pleiotropic effects. Insulin signaling pathways are the most important biochemical pathways. They are involved in the regulation of some important processes, such as glucose and lipid metabolism, protein synthesis, cell proliferation, cell growth, differentiation, and apoptosis. Insulin promotes glucose homeostasis and metabolism. It regulates blood glucose levels and stimulates the storage of glucose as glycogen in organs such as the liver, muscles, and adipose tissue. Insulin also facilitates glucose transport into cells through the translocation of GLUT4 from intracellular compartments into the cell membrane [1,2].

Insulin exerts its effects by binding to the insulin receptor (INSR), located on the plasma membrane of target cells. Insulin binds to the INSR that belongs to the receptor tyrosine kinase superfamily, initiating the intracellular signaling pathway. There are two primary insulin signaling pathways. An activated receptor can phosphorylate a number of intracellular substrates, such as insulin receptor substrate (IRS) protein, or SH3-containing protein (SHC), which are associated with signaling pathways. The activation of phosphatidylinositol-3-kinase (PI3K), due to phosphorylation of tyrosine in some substrates, causes the activation of the serine/threonine-specific protein kinase B (AKT). The activation of INSR by insulin also initiates the second primary pathway, the mitogen-activated protein kinase (MAPK) pathway. The insulin signaling pathway may differ in dependence on role of insulin in particular tissues/organs. Normal fasting insulin levels range between 2.55 and 18.4 μIU/mL. Concentrations below 2.55 μIU/mL indicate hypoinsulinemia, whereas levels above 18.4 μIU/mL indicate hyperinsulinemia. Both reduced and excessive insulin levels have been associated with pathological conditions, including cancers [3], type 2 diabetes mellitus, cardiovascular disease, metabolic syndrome, and hypertension [4]. Furthermore, hyperinsulinemia contributes to the development of neurodegenerative diseases [5,6,7]. Performed clinical and basic research revealed the role of hormones in the pathogenesis of numerous diseases. On the other hand, many aspects of these associations require further investigations [1].

## 2. The Role of Insulin in the Human Central Nervous System

In medical textbooks published over 30 years ago, the brain was described as an “insulin insensitive organ” [6]. However, subsequent observations revealed the presence of insulin, as well as its receptors in the CNS [8,9,10]. While insulin was traditionally believed to be produced exclusively by pancreatic β-cells, more recent studies have identified low levels of insulin in certain CNS neurons [11]. Its levels in the cerebrospinal fluid (CSF) are much lower as compared to the plasma [12]. Obtained results suggest that the majority of insulin found in the brain is derived from the circulation of insulin secreted by pancreatic β-cells [13]. Molecules smaller than ~400 Da can diffuse passively across the blood–brain barrier (BBB). With a molecular weight of 5.8 kDa, insulin is too large to cross via passive diffusion. Therefore, its crossing of the BBB is associated with active transport mechanisms to enter the brain. The crossing of insulin via the BBB from the circulation to the brain is associated with the presence of the capillary endothelial cells of the BBB and is due to a selective, saturable, receptor-dependent mechanism [14,15]. It has also been proposed that insulin may bypass the BBB through direct neuroanatomical connections between the olfactory nerves and the brain [16].

### 2.1. Insulin Signaling Pathway in the Healthy Brain

Insulin plays several distinct roles in the brain. It regulates the metabolism of glucose, proteins, and lipids; contributes to neural development and neuronal activity; and participates in learning and memory processes. It is also involved in glucose and energy homeostasis [17]. Insulin enhances cognitive functions, including learning and memory, and particularly verbal memory [18]. For cognitive function, insulin signaling in the limbic system and the hypothalamus is very important [19]. These functions may be associated with the modulation of concentrations of neurotransmitters involved in cognition by insulin, such as acetylcholine [20]. Beyond metabolic regulation, insulin influences brain-specific processes, including synaptic plasticity, age-related neurodegeneration, executive function, apoptosis, cerebral blood flow, and glial inflammatory responses. Its role is also associated with β-amyloid (Aβ) peptide clearance and tau protein phosphorylation. It also regulates neurotransmission. This action is due to the modulation of glutamatergic and GABAergic receptor trafficking, processes that are central to synaptic plasticity and memory formation [21].

Two principal insulin signaling pathways mediate its functions in the brain. The PI3K/AKT pathway regulates cell growth, differentiation, neuronal survival, learning, memory, and synaptic plasticity. The MAPK pathway, in turn, is primarily involved in memory formation and gene expression [5,22,23].

#### 2.1.1. Synthesis of Insulin

Humans possess a single insulin gene, *INS*, located on chromosome 11 [24]. Insulin is synthesized primarily in the β-cells of the islets of Langerhans in the pancreas. Translation initially produces preproinsulin, which is processed in the rough endoplasmic reticulum. A signal peptidase cleaves the signal sequence, converting preproinsulin into proinsulin. The proper folding and stabilization of proinsulin, involving linkage of the semihelical A domain and helical B domain, are achieved through three disulfide bounds. The next step, which occurs in the Golgi apparatus, is associated with sorting of proinsulin into secretory granules. Prohormone convertases PC1/3 and PC2 cleave the C-peptide in the proinsulin. Then C-terminal basic amino acids are removed from the peptide chains by carboxypeptidase E, resulting in mature insulin. The final hormone consists of A- and B-chains linked by disulfide bonds [25].

#### 2.1.2. Characteristics of Insulin Receptor

**Figure 1:**
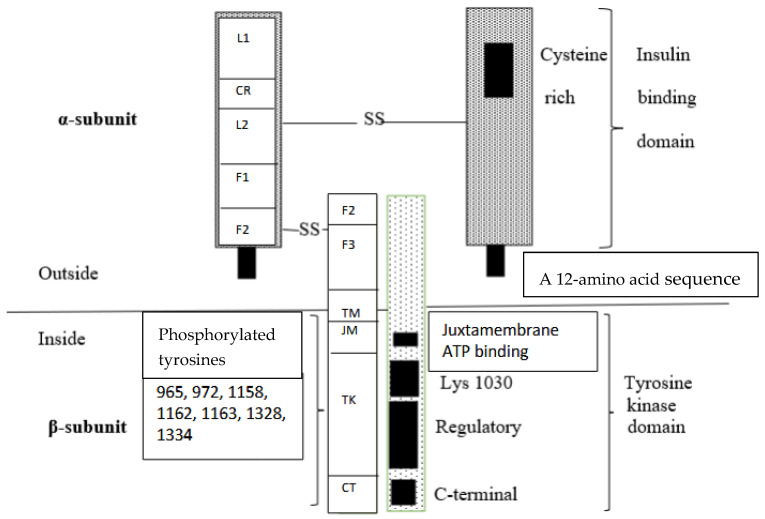
The INSR is composed of two extracellular α-subunits, which serve as the insulin-binding site, and two β-subunits, which mediate signal transduction. Isoform B of INSR also contains a 12-amino acid sequence, whereas isoform A lacks this sequence. These subunits are linked by a single disulfide bond. Conformational changes in the α-subunits, caused by insulin binding to the INSR, enable the binding of ATP to the β-subunit. The binding of ATP activates a tyrosine kinase in the β-subunits, causing autophosphorylation of the receptor. The phosphorylated INSR, in turn, phosphorylates other proteins, which function as insulin receptor substrates. Abbreviations: CR—cysteine-rich domain, CT—C-terminal region of the β-subunit, F—fibronectin type III domain, JM—juxtamembrane, L—leucine-rich region, SS—disulfide bridges, TK—tyrosine kinase, TM—transmembrane.

INSRs are widely distributed in the brain, with the highest density observed in the olfactory bulb, hypothalamus, hippocampus, cerebral cortex, and cerebellum [26]. The high level of INSRs in the hippocampus, entorhinal cortex, and frontal cortex suggests that insulin is involved in learning and memory [27]. The INSR originates from a single polypeptide precursor (preproreceptor), which undergoes post-translational cleavage by a furin-like proteolytic enzyme into two subunits, α and β. These subunits are subsequently glycosylated, folded, and linked by disulfide bridges, forming a functional homodimer (α2 β2), which is a mature receptor [28,29,30,31]. The two α-subunits are connected by disulfide bridges within the first and second fibronectin type III domains, while the α and β subunits are linked by additional disulfide bonds (Figure 1) [32,33,34,35,36].

Structurally and genetically, the INSR belongs to class II receptor tyrosine kinases [37]. It is a heterotetrameric membrane glycoprotein consisting of two α- and two β-subunits. Insulin binds to the extracellular α-subunit, which induces changes leading to dimerization and activation of the intracellular β-subunit. Autophosphorylation of tyrosine residues within the β-subunit occurs through the intrinsic tyrosine kinase activity of the receptor. The autophosphorylated residues play a role as the binding sites for signaling receptor substrates, which contain Src homology 2 (SH2) domains. The kinases also phosphorylate these domains, and the activation of these domains may be caused by conformational changes, starting the intracellular cascade of signal transduction [35].

In humans, there are two isoforms of INSR. The INSR mRNA undergoes alternative splicing of exon 11 that codes a 12-amino acid sequence in the C-terminal part of the α-subunit [32,38]. Isoform A (INSR-A) lacks this 12-amino acid sequence, whereas isoform B (INSR-B) contains it. In the CNS, neurons express the INSR-A, whereas glia predominantly express INSR-B. Of note, in the periphery, the majority of INSR expression is represented by the INSR-B isoform. The INSR-A isoform has approximately a 1–2-fold higher affinity for insulin, as compared to the INSR-B isoform [39]. INSR-A is predominantly expressed in fetal tissues and in the adult nervous system, and INSR-B is expressed mainly in adipose tissue, the liver, and skeletal muscle [40,41]. The binding of insulin to INSR causes a large conformational change in the receptor. This change facilitates autophosphorylation of INSR and triggers the downstream insulin signaling pathway [32]. The β-subunit in its cytoplasmic region contains three domains: a juxtamembrane domain, a tyrosine kinase domain, and a carboxy-terminal domain. Sites of tyrosine autophosphorylation have been mapped in all three regions [42,43]. Substrates involved in the insulin signaling pathway may interact with each domain, but phosphorylation of the three sites within the kinase domain is important for activation. Observations have revealed that phosphorylated kinase domains, together with the juxtamembrane domain, enhance transphosphorylation and activation [2]. The insulin-activated INSR kinase induces autophosphorylation of multiple tyrosine residues in the intracellular region: phosphor-Y953 (pY953) and pY960 in the juxtamembrane domain; pY1146, pY1150, and pY1151 in the kinase domain; and pY1316 and pY1322 in the C-terminal domain [32,43,44,45]. Phosphorylation of these tyrosine residues provides docking sites for effector and adaptor proteins, leading to phosphorylation-mediated signaling cascades [46]. INSR may also be activated by the binding of insulin-like growth factor-1 (IGF-1) [2], and IGF-1 receptor (IGF-1R) may be activated by insulin. Initiation of the insulin signaling pathway occurs upon insulin binding to INSR.

#### 2.1.3. Insulin Receptor Substrates

To date, at least nine intracellular substrates of INSR and IGF-1R have been identified. These substrates belong to the insulin/IGF-1 receptor substrate (IRS) protein family [47,48,49,50]. There are also other substrates including GAB-1, DOK1, CBL, SH2B2, SHP2, and various isoforms of Shc [2,51]. In each substrate, tyrosine residues occur within specific sequence motifs and undergo phosphorylation. The IRS proteins, Shc, SH2B2, and CBL, are the best characterized. Each of these docking proteins contains specific domains—IRS proteins and Shc contain a phosphotyrosine-binding (PTB) domain, while SH2B2 and CBL contain an SH2 domain (SH2B2, CBL), both of which are involved in receptor interaction [2]. SH2 domains recognize and bind to phosphorylated tyrosine residues on receptor proteins, particularly in receptor tyrosine kinases (RTKs). PTB domains bind to phosphorylated tyrosine residues and in a specific sequence context, such as NPXpY/NXXpY. NPXY motifs of PTB are different from the typical SH2 domain-binding sites. There are six members of the IRS proteins family, classified as IRS-1, IRS-2, IRS-3, IRS-4, IRS-5 (also known as DOK4), and IRS-6 (also known as DOK5) [52]. It is notable that IRS-5 and IRS-6 are DOK (downstream of tyrosine kinase/docking protein), not classical IRS proteins. There are seven members of the DOK family (DOK1-7), which play an important role in the receptor tyrosine kinase signaling pathway as regulatory proteins. DOK4 and DOK5 are positive regulators of the MAPK pathway. DOK proteins have similar domain architectures. Based on sequence homology and functional interaction, they can be distinguished from the IRS family. In humans, three homologous IRS proteins are expressed: IRS-1, IRS-2, and IRS-4. The *IRS-3* gene is a pseudogene in humans, and therefore no protein is synthesized, while it is functional in mice [53]. IRS-1 and IRS-2 are widely distributed, whereas IRS-4 expression is more limited and tissue-specific [54]. IRS-4 is expressed in tissue-specific manner, predominantly in the brain, thymus, kidney, skeletal muscle and embryonic tissues. Its physiological function still remains elusive. It plays an important role in cell proliferation and in liver regeneration. Unfortunately, its overexpression is observed in several cancers. IRS-5 and IRS-6 have limited expression and functions. IRS-5 is expressed in the kidney and liver. It activates the MAPK pathway. IRS-6 has short structure and a reduced number of phosphorylation sites; therefore, it has limited signaling capacity. Most insulin effects are associated with the interaction of IRS-1, IRS-2, and Shc with the INSR [51,55].

#### 2.1.4. The PI3K/AKT Signaling Pathway (Figure 2)

Phosphatidylinositol 3 kinase (PI3K) is a serine/threonine lipid kinase. It contains two domains: a catalytic subunit (p110), which has three isoforms, and a regulatory subunit (p85), which has eight isoforms [34,56]. Tyrosine phosphorylation of IRS proteins within specific motifs begins the PI3K/AKT signaling pathway. The activation of this cascade occurs through the binding of the p85 regulatory subunit to IRS through its two SH2 domains [57], resulting in the activation of the catalytic domain. The activated p110 domain rapidly phosphorylates phosphatidylinositol 4,5-bisphosphate (PIP2), generating the lipid second messenger phosphatidylinositol (3,4,5) triphosphate (PIP3), which recruits 3-phosphoinositide-dependent protein kinase-1 (PDK1), causing its activation. The activated PDK-1 phosphorylates AKT, also referred to as protein kinase B (PKB), at threonine 308 [58]; AKT is subsequently phosphorylated at serine 473 in its C-terminal region by mTORC2 [59]. AKT, originally identified as an oncogene from the Akt8 retrovirus, is a serine/threonine-specific protein kinase [60]. There are three subtypes of AKT: AKT1, expressed in most tissues; AKT2, located in muscles and adipose tissue; and AKT3, predominantly expressed in brain cells; however, its expression is also detected in other organs and tissues [7,61]. AKT regulates numerous cellular activities, including growth, proliferation, adhesion, neovascularization, and apoptosis [62]. It can enhance cell survival [63] and plays an important role in neuron functions, such as metabolism, transcription, protein synthesis, proliferation, growth, and survival [7].

There are four critical downstream substrates of AKT: mammalian target of rapamycin (mTOR), glycogen synthase kinase 3 (GSK-3), forkhead box-containing protein, O subfamily transcription factors (FOXO), and AKT substrate of 160 kDa (AS160) [33]. mTOR is a serine/threonine kinase involved in the regulation of protein synthesis [64]. It is the main target of rapamycin, a macrolide antibiotic [7]. There are two complexes which contain mTOR, mTORC1 and mTORC2, where C is a complex, that is a product of reaction with a diverse range of proteins. It is a serine/threonine kinase, acting as a nutrient sensor. mTOR is involved in the protein synthesis, causing phosphorylation of eukaryotic translation initiation factor 4E-binding protein 1 (4EBP1) and p70 ribosomal protein S6 kinase (p70S6K). It has also been observed that the mTOR pathway plays a role in neuronal and synaptic plasticity, which underlies learning and memory performance [65,66]. Based on current evidence, it has been suggested that the activation of mTOR may enhance synaptic plasticity and memory formation [7].

Glycogen synthase kinase (GSK) is a serine/threonine protein kinase that inhibits glycogen synthase and is involved in several cellular processes, playing a role in multiple signaling pathways [67]. There are two isoforms of GSK-3: GSK-3α, and GSK-3β [68]. The GSK-3β isoform is associated with insulin signaling, glycogenesis, neutrophic factor signaling, Wnt signaling, neurotransmitter signaling, and microtubule assembly [69].

FOXO, particularly FOXO1, is a nuclear transcription factor which is excluded from the phosphorylation of AKT. This family of transcription factors controls the expression of lipogenic and gluconeogenic genes. FOXO1 stimulates the expression of several genes, such as phosphoenolopyruvate carboxykinase (PEPCK), an important enzyme in gluconeogenesis [55] and cyclin G2, which causes the arrest of cell cycle and other genes involved in insulin-induced mitogenesis [70]. Following the phosphorylation by AKT, FOXO1 is sequestered in the cytoplasm.

**Figure 2:**
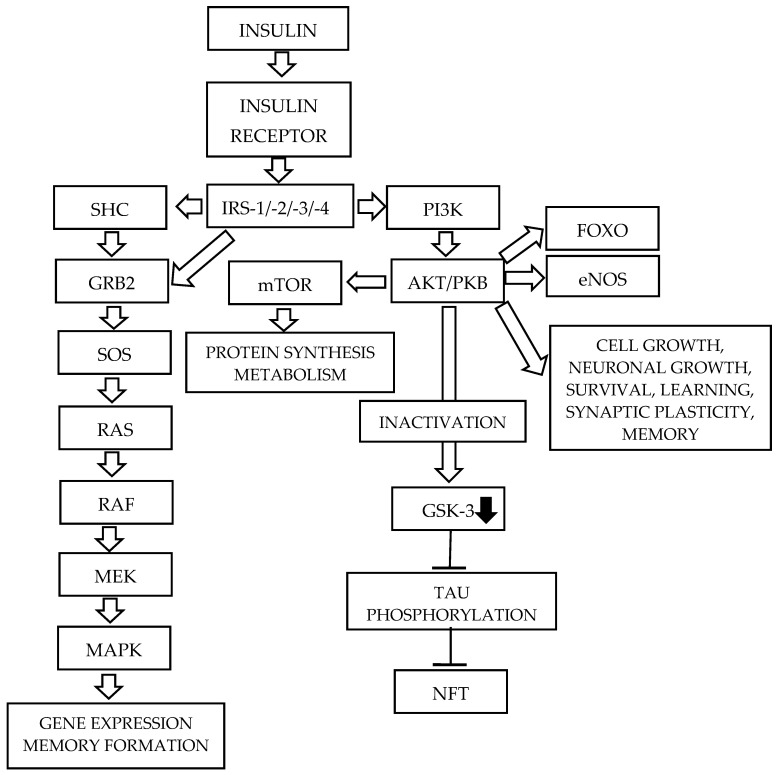
The insulin signaling pathway in a healthy human brain [5]. Insulin exerts its effect by binding to the insulin receptor. There are two primary insulin signaling pathways. An activated receptor can phosphorylate a number of intracellular substrates, such as insulin receptor substrate proteins or SH3-containing protein, associated with signaling pathways. The activation of PI3K, due to the phosphorylation of tyrosine in some substrates, activates the serine/threonine-specific kinase B. An activated insulin receptor by insulin also initiates the second primary pathway, the mitogen-activated protein kinase. These two insulin signaling pathways are activated independently; however, they may crosstalk. For further details, see the text. Abbreviations: AKT/PKB—Serine/threonine protein kinase, also known as protein kinase B; GSK-3—Glycogen synthase kinase 3; eNOS—Endothelial nitric oxide synthase; FOXO—Forkhead box O; IRS-1/-2/-3/-4—Insulin receptor substrate—(1, 2, 3, 4); GRB2—Growth factor receptor-bound protein 2; MAPK—Mitogen-activated protein kinase; MEK—MAPK/ERK kinase; mTOR—mammalian target of rapamycin; NFT—Neurofibrillary tangle; PI3K—Phosphatidylinositol 3 kinase; RAF—Rapid accelerated fibrosarcoma; RAS—Rat sarcoma; SHC—SH3-containing protein; SOS—Son-of-sevenless; Tau —Microtubule-associated protein (MAP) tau (MAPT).

AS160, also called TBC1D4, is a 160 kDa AKT substrate that is involved in insulin-stimulated glucose transport. AS160 is a GTPase-activating protein. After its phosphorylation, AS160 regulates the activity of small G proteins known as RAB. These proteins are important for the regulation of membrane trafficking, through the exchange of GTP for GDP [71,72,73] (Figure 2).

There are also multiple other substrates of AKT associated with insulin action.

#### 2.1.5. The MAPK Signaling Pathway (Figure 2)

A second essential component of the insulin signaling pathway is the mitogen-activated protein kinase (MAPK) pathway, also called the GRB2-SOS-RAS-MAPK pathway (Figure 2). This signaling pathway is activated independently of the PI3K/AKT signaling pathway; however, they may crosstalk. INSR and IRS proteins contain docking sites for adaptor molecules with SH2 domains, such as growth factor receptor-bound protein 2 (GRB2) and Src homology 2 domain-containing (SHC). GRB2 is an adaptor protein that binds to IRS and SHC. GRB2 exists in a complex with son-of-sevenless (SOS), a guanine nucleotide exchange factor (GEF) for the small G-protein RAS [74]. SOS catalyzes the switch of membrane-bound RAS from an inactive, GDP-bound form (RAS-GDP) to an active, GTP-bound form (RAS-GTP) [75]. The activation of RAS may also be facilitated by GTPase-Activating Proteins (GAPs). The active form of GTP-RAS interacts with and stimulates downstream effector molecules, such as the serine/threonine kinase RAF, which phosphorylates and activates MAPK/ERK kinase 1 and 2 (MEK1 and MEK2). MEK1/2 are dual-specificity tyrosine/threonine protein kinases. MEK1 and MEK2 phosphorylate and activate the MAP kinases Extracellular Signal-Regulated Kinases 1 (ERK1) and 2 (ERK2). Activated ERK1 and ERK2 are translocated to the cell nucleus, where they phosphorylate and transcriptionally activate transcription factors such as ELK. The MAPK signaling pathway is associated with cell division, proliferation, differentiation, protein synthesis, and cytoskeletal reorganization [2,35,76,77]. ERK is coupled to the cAMP response element-binding protein (CREB) and activates it. ERK also activates CREB indirectly by the p90 ribosomal S6 kinase (RSK). Its activation induces brain-derived neurotrophic factor (BDNF)-induced long-term potentiation at dentate gyrus synapses [78]. The activation of MAPK signaling linked with CREB increases the transcription of specific genes associated with neuronal survival [79]. For more details, see [2,7,28,32,80,81].

## 3. Alzheimer’s Disease

Alzheimer’s disease (AD) is a devastating and gradually progressive disorder. It is the most common cause of dementia [82], accounting for over 60% of all cases [83]. To date, over 100 types of dementia have been described. One of these is AD, the best-known form [84]. AD is characterized by memory loss, cognitive decline, and difficulties with daily activities [85]. The first symptom is usually gradual memory loss; however, other signs may also occur, such as difficulty finding the right word, recognizing familiar people, or solving problems [86,87,88]. AD causes progressive brain degeneration, associated with selective neuronal loss, reduction of dendritic spines and synapses, impaired neurotransmission, and increasing isolation of remaining nerve cells [89].

There are two forms of AD: familial or early-onset AD (fAD), which constitutes less than 5% of all cases. It is diagnosed before the age of 65 and is associated with autosomal dominant mutations in three major genes: amyloid precursor protein (APP), presenilin 1 (PS1), and presenilin 2 (PS) [90]. The second form of AD is sporadic or late-onset AD (sAD), which accounts for the vast majority of cases (approximately 95–99%). sAD lacks a well-defined etiology, but it is suggested that genetic, environmental, behavioral, and metabolic factors contribute to its development [91]. Sporadic AD is usually diagnosed after the age of 65, with advanced age being the most prominent risk factor. The most significant genetic risk factor for sAD is a variation in the apolipoprotein E (*APOE-ε*) gene, particularly the APOE isoform. APOE is a lipoprotein secreted by astrocytes that modulates Aβ transport and lipid/cholesterol metabolism [92,93]. It is also localized throughout the body, including the liver, adipose tissue, and other organs and tissues. There are three isoforms of APOE: APOE-ε2, APOE-ε3, and APOE-ε4 [94]. APOE-ε4 increases the risk of dementia [95] and is involved in the formation of caveolae, which modulates insulin signaling through its receptor. This action promotes insulin resistance, leading to impaired insulin signaling, mitochondrial respiration, and glycolysis, ultimately contributing to dementia. Increased caveolae formation also enhances the Aβ fibrils deposition [96]. APOE plays a role in cholesterol and Aβ homeostasis [97,98]. The presence of two *APOE-ε4* alleles increases AD risk 12-fold, while one allele increases it 3.7-fold [99]; however, the exact risk fold numbers for *APOE-ε4* carriers are approximate and population dependent. *APOE-ε4* is found in approximately 50–60% of AD patients, suggesting that additional factors contribute to disease development [89]. AD is also associated with brain insulin/IGF-1 deficiency and brain/IGF-1 resistance. Therefore, it has been suggested that AD represents a brain-specific form of diabetes mellitus, often referred to as Type 3 Diabetes Mellitus (T3DM) [29,100,101].

### 3.1. Molecular Pathophysiology of Alzheimer’s Disease

Results obtained in studies have revealed that in the development of AD, pathophysiological changes may occur up to 20–30 years before clinical symptoms [102]. Neuropathologically, AD is associated with the deposition of extracellular β-amyloid (Aβ40 and Aβ42) in the brain parenchyma, forming neuritic (senile) plaques (SPs); the presence of Aβ42 oligomers; β-amyloid angiopathy around cerebral blood vessels; and intraneuronal deposits of hyperphosphorylated, abnormally conformed, and truncated tau protein in the form of neurofibrillary tangles (NFTs) [103,104].

#### 3.1.1. The Role of APP and Aβ in Development of AD

An impairment of synaptic functions initiates the pathogenesis of AD. This pathology is due to the accumulation of Aβ, produced from APP. The gene encoding APP is located on chromosome 21 [105,106] and exists in three isoforms: APP695, APP751, and APP770 [107]. Individuals with trisomy 21 (47, XX +21, or 47, XY +21), known as Down’s syndrome, have an increased risk of developing AD, caused by an additional copy of the APP gene, resulting in its overexpression [108]. An acute noxious stimulus may trigger a pathophysiological cascade that causes the disruption of APP metabolism. Aβ dysregulates intracellular Ca^2+^, inducing neurofibrillary tangle formation and neuronal cell death [109]. Therefore, Aβ may act as a triggering factor in the development of both forms of AD: fAD and sAD [110]. APP is a transmembrane widely expressed protein, especially in the CNS, and is most abundant in synapses. APP functions as a cell surface receptor, contributing to the regulation of synapse formation, neural plasticity, antimicrobial activity, and iron export [5]. Along with amyloid precursor-like protein 2 (APLP-2), APP is involved in maintaining learning and memory [111]. There are two different pathways associated with the processing of APP: 90% is performed by the non-amyloidogenic pathway (nonplaque-forming or secretory) and the remaining 10% by the amyloidogenic pathway (plaque-forming). Insulin promotes the non-amyloidogenic processing of APP, and disturbances in insulin signaling may increase pathological amyloid-β accumulation [112]. In the non-amyloidogenic pathway, APP is cleaved by α-secretase, producing a C-terminal fragment α (CTFα, C83) and a soluble N-terminal fragment α (sAPPα). CTFα is subsequently cleaved by γ-secretase, generating a smaller C-terminal fragment (C3) [113,114]. In this process, α-secretase prevents the generation of Aβ peptide. In the amyloidogenic pathway, which occurs in an acidic environment, β-secretase cleaves APP into a smaller N-terminal (sAPPβ) fragment and a longer C-terminal fragment (CTFβ, C99) containing the full amyloidogenic sequence. Subsequent cleavage of CTFβ is performed by γ-secretase formation of an APP intracellular cytoplasmic domain (AICD) and Aβ fragments. These fragments are released into the extracellular environment, where Aβ fragments rapidly form oligomers, prefibrillary aggregates, and fibrils, ultimately forming β-amyloid plaques [113,114]. Aβ peptides consist of 38 (Aβ38), 40 (Aβ40), and 42 (Aβ42) amino acids. Increased APP expression, combined with altered proteolysis, leads to the accumulation of Aβ40 and Aβ42. In fAD mutations and the inheritance of the APOE-ε4 allele, alter γ-secretase, increasing Aβ42 production [115]. These mutations promote Aβ peptides’ synthesis and deposition in the brain. The mechanisms of Aβ accumulation in sAD are actively investigated [116,117]. In the healthy brain, neuronal activity is associated with the Aβ release into the extracellular space, and local proteases regulate its levels [118]. Native APP facilitates memory and learning via synaptic activity and dendritic spine formation [107]. Amyloid plaques block signaling pathways and disrupt cell connections, causing neuronal death. Animal studies have shown that intracellular administration of Aβ leads to a memory deficit similar to those observed in AD patients. Memory and learning impairments are associated with disrupted synaptic plasticity, caused by intraneuronal Aβ accumulation [119]. The deleterious effects of Aβ on neurons are due to an immune inflammatory reaction in glial cells, leading to the phagocytosis of neuronal and synaptic structures [120]. In rodents, hippocampal administration of Aβ oligomers induces synaptic loss and neuronal dysfunction, which can result in memory impairment [121]. Intracerebroventricular administration of Aβ in non-human primates produces behavioral changes and AD-like pathology [122]. Insulin plays an important role in regulating the APP metabolism. This hormone is involved in the control of the balance between Aβ Aβ anabolism and catabolism. Decreased insulin levels or impaired insulin action may lead to increased NFT formation, oxidative cell damage, elevated Aβ levels, and amyloid plaque in the brain [123]. 

#### 3.1.2. The Role of Insulin Degrading Enzyme in the Development of AD

Insulin-degrading enzyme (IDE) is a thiol zinc-metallo-endopeptidase that cleaves insulin, Aβ, glucagon, and calcitonin. It is involved in the regulation of insulin and Aβ levels in the brain. IDE has a higher affinity for insulin than for Aβ and at high insulin levels, Aβ competes for IDE, increasing the Aβ deposition [124]. Low levels of Aβ upregulate IDE; therefore, IDE may serve as an important therapeutic target due to its role in the degradation of Aβ and other substances [125]. This observation needs further investigation, because the regulation of IDE by Aβ is mostly preclinical and not fully established in humans. The activation of insulin signaling in the CNS upregulates IDE activity and may correct the IDE defects observed in AD [126]. Results obtained in performed studies revealed that insulin prevents the formation of Aβ fibrils, stimulates the internalization of Aβ oligomers, and inhibits their binding to neurons. In this way, insulin protects synapses against Aβ oligomers [127]. But these protective effects of insulin are mainly experimental. IR impairs the protective role of insulin against Aβ accumulation, and Aβ deposits downregulate insulin expression. Studies have shown that Aβ peptides inhibit insulin binding to its receptors and decrease receptor autophosphorylation, resulting in impaired insulin signaling [128]. Brain IR, an early and common feature of AD, is associated with IRS-1 dysfunction mediated via serine phosphorylation, induced by Aβ oligomers, which contributes to cognitive decline [129].

#### 3.1.3. The Role of Tau Protein in Development of AD

For many years, it has been discussed whether tau protein is involved in AD pathology. Results suggest that when tau protein assumes pathological forms, it compromises neuronal function and promotes cell death [130]. The human tau gene is located on chromosome 17 [110]. In the adult human brain, six tau isoforms are expressed, resulting from alternative splicing [131]. Tau protein is classified as a microtubule-associated protein (MAP) [132]. It participates in the assembly and stabilization of microtubules, which are involved in several cellular processes, including cell morphogenesis, cell division, and intracellular trafficking, and it also influences synapses and neuronal nuclei [133], from which it is released into an extracellular environment [134]. Tau protein plays important roles in neurogenesis and synaptic integration [135,136], neuronal maturation [137], neuronal development and synaptogenesis [138], normal myelination [139], and synaptic plasticity [140]. Tau also contributes to behavior [141], learning, memory [142], and other processes [110]. Normal brain function requires tau protein. Beside these functions, tau deletion leads to brain iron accumulation, contributing to conditions such as Parkinson’s disease [143]. In AD, all six tau isoforms may be abnormally phosphorylated, resulting in the formation of neurofibrillary tangles and destabilization of the microtubule network [144]. Normal tau is a phosphoprotein and requires phosphorylation to exert its function. However, rendering tau insoluble reduces its affinity for microtubules, causing neurodegeneration [145]. Results suggest that the soluble form of tau is also the most cytotoxic constituent, causing synaptic deficits in AD, rather than the neurofibrillary tangles associated with its aggregated form [110]. In AD brains, tau is hyperphosphorylated approximately three-fold compared to normal brains [113]. Out of 85 phosphorylable residues, more than 40 phosphorylation sites have been detected in tau from AD brains, with 28 sites being exclusively phosphorylated [114]. In vitro studies have shown that more than 30 kinases can phosphorylate tau. These kinases include cyclin-dependent kinase 5 (DK5), glycogen synthase kinas 3β (GSK3β), MAPK1, ERK2, c-Jun N-terminal kinase (JNK), and others. Tau hyperphosphorylation may also result from decreased dephosphorylation, mediated by protein phosphatases (PPs), such as PP1, PP2, PP2B, and PP5 [146,147]. Imbalances between kinases and phosphatases lead to tau hyperphosphorylation; therefore, a balance between tau kinases and phosphorylases is necessary to maintain homeostasis. Hyperphosphorylation induces conformational changes in tau, impairing its ability to bind microtubules. Misfolded tau monomers cannot be transported into the axon, which is necessary for accumulation, oligomerization, and aggregation in neuronal perikarya. Impaired aggregation leads tau to accumulate in β-sheet-rich domains and form filaments. Aggregated tau may be deposited in NFTs and is associated with tauopathies. Animal studies in AD models revealed that tau is important for regulating brain insulin signaling [148], and its deletion impairs the hippocampal response to insulin. Loss of tau function contributes to cognitive and metabolic impairments in AD patients [148]. In AD patients, insulin is accumulated as oligomers in neurons with hyperphosphorylated tau. Insulin accumulation correlates with tau phosphorylation levels, promoting tauopathy progression. This accumulation is also associated with insulin resistance and reduced INSR levels [149].

### 3.2. Impaired Insulin Signaling Pathway as a Cause of Alzheimer’s Disease (Figure 3)

The exposure of cells to chronically elevated insulin levels causes partial downregulation of insulin signaling. Continuous hyperinsulinemia, a characteristic feature of IR, impairs insulin signal transduction due to receptor dysfunction, rather than decreased INSR levels. Chronic hyperinsulinemia also impairs insulin signaling mainly via IRS-1 serine phosphorylation, not only receptor desensitization. Prolonged hyperinsulinemia diminishes INSR autophosphorylation compared to short-term insulin exposure, resulting in impaired PI3K/AKT signaling [150,151]. In muscle and adipocytes, impaired PI3K/AKT signaling reduces GLUT4 translocation from intracellular compartments to the cell membrane, decreasing glucose uptake. In CNS, GLUT1 and GLUT3 play an important role in glucose uptake. Neuronal health, synapse formation, and brain development depend on insulin and glucose metabolism. Observations have shown that Aβ oligomers block the PI3K/AKT pathway, leading to neuronal death. Post-mortem examination of AD brain revealed decreased insulin and INSR levels [152], reduced AKT and GSK-3β phosphorylation, and lower PI3K subunits (p85 and p110) expression. Similar reductions were observed for IGF-1 and IGF-1R [153]. Insulin signaling may also be activated via IGF-1R, as well as by INSR/IGF-1R hybrid receptor, and IGF-1 can bind INSR, activating the insulin signaling pathway. Impaired signaling suppresses PI3K/AKT signaling [152]. Phosphorylation of Ser9 by AKT causes its activation. Active GSK-3β induces tau hyperphosphorylation, promoting apoptotic neuronal death. Reduced insulin levels decrease GSK-3β phosphorylation, leading to its activation. Animal studies in AD rat models have shown that acute insulin treatment improve memory functions impaired by insulin dysregulation [123].

In several cell signaling pathways, GSK-3 plays an important role, especially GSK-3β, which is essential for processes such as insulin signaling, microtubule assembly, neurotransmitter signaling, gluconeogenesis, and others. The N-terminal regulatory regions of both GSK-3 isoforms, GSK-3α and GSK-3β, are phosphorylated by AKT. Inactivation of GSK-3 improves glucose metabolism. It also plays an important role in regulating apoptotic processes. Inactivated GSK-3 enables the regulation of metabolic activities and transcription factors, promoting cell development and extending neuronal cell lifespan [69].

**Figure 3:**
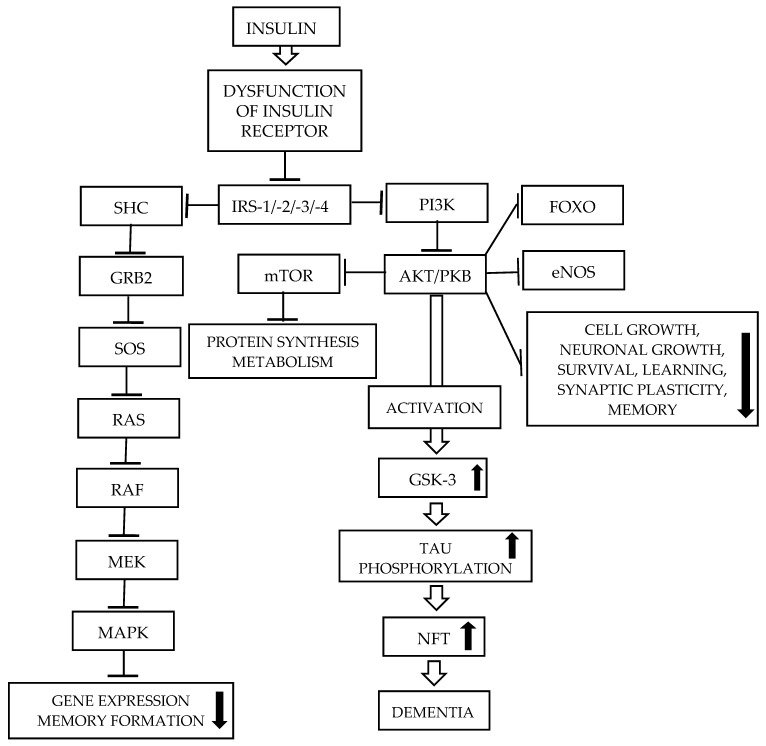
Changes in insulin signaling pathways in the brains of AD patients due to brain IR [5]. Chronically elevated insulin levels cause partial downregulation of insulin signaling. Continuous hyperinsulinemia impairs insulin signal transduction due to receptor dysfunction. Chronic hyperinsulinemia also impairs insulin signaling mainly via IRS-1 serine phosphorylation, not only receptor desensitization. For further details, see the text. Abbreviations: AKT/PKB—Serine/threonine protein kinase, also known as protein kinase B; GSK-3—Glycogen synthase kinase 3; eNOS—Endothelial nitric oxide synthase; FOXO—Forkhead box O; IRS-1/-2/-3/-4—Insulin receptor substrate—(1, 2, 3, 4); GRB2—Growth factor receptor-bound protein 2; MAPK—Mitogen-activated protein kinase; MEK—MAPK/ERK kinase; mTOR—mammalian target of rapamycin; NFT—Neurofibrillary tangle; PI3K—Phosphatidylinositol 3 kinase; RAF—Rapid accelerated fibrosarcoma; RAS—Rat sarcoma; SHC—SH3-containing protein; SOS—Son-of-sevenless; Tau—Microtubule-associated protein (MAP) tau (MAPT).

It should be noted that activated GSK-3 promotes tau phosphorylation and Aβ formation. Therefore, it is suggested that blocking the phosphorylation that activates GSK-3β may reduce AD pathology and represent a promising therapeutic strategy [154].

AMP-activated protein kinase (AMPK) is a metabolic serine/threonine protein kinase that functions as an “energy receptor” and is involved in restoring ATP concentrations by decreasing anabolic processes or increasing catabolic processes [4,155]. It regulates several processes, such as the synthesis of fatty acids, triglyceride, and protein, as well as glucose transport, cellular growth and proliferation, and mitochondrial functional biogenesis. AMPK negatively regulates proteins central to ATP-consuming processes, such as the transducer of regulated CREB activity 2 (TORC2), glycogen synthase, and sterol regulatory element-binding proteins (SREBP). The activation of AMPK downregulates or inhibits gluconeogenesis, and the synthesis of glycogen, lipids, and proteins [156,157]. IR suppresses AMPK activity through the overactivation of AKT. The inhibition of AMPK activity allows Aβ oligomers to reduce surface levels of glucose transporters (GLUTs) in hippocampal neurons, resulting in IR [158]. IR can increase amyloidogenesis by suppressing the insulin-PI3K/AKT signaling pathway [152], leading to the upregulation of GSK-3β activity. However, increased AMPK activation stimulates IRS phosphorylation, causing the inhibition of GSK-3β and thus the activation of insulin signaling [159,160]. The activation of AMPK can stimulate the expression of GLUT1 and GLUT4, increasing the rate of glucose utilization, causing the synthesis of large amounts of ATP, and thereby improving neuronal activity and homeostasis [161]. With regard to tau phosphorylation, the role of AMPK is still debated [162]. Abnormally increased levels of phosphorylated AMPK were detected in the cytoplasm of cortical neurons in the AD brain, as well as in other tauopathies. Phosphorylated AMPK and phosphorylated tau strongly co-localize in pre-tangle-and tangle-bearing neurons in most brain regions affected by tau pathology in AD patients [163]. In vitro observations revealed that AMPK is a tau kinase that can phosphorylate tau at sites within the microtubule-binding domain and the flanking regions [162]. AMPK may contribute to reducing tau levels and tau phosphorylation various downstream mechanisms [156,161]. For more details, see [155]. It was also observed that dysregulated AMPK is associated with the onset and progression of AD [156,157,164]. There are conflicting and controversial results on the association of AMPK with AD; therefore, this issue requires further investigation.

The MAPK signaling pathway may also be associated with pathologies observed in AD, such as tau phosphorylation, excitotoxicity, and synaptic plasticity. Post-mortem investigations revealed that the brains of AD patients have elevated immunoreactivity at the phosphor-p38 MAPK level. MAPK is also involved in the development of NFTs in neuronal locations and the build-up of Aβ plaques [165]. Therefore, it is suggested that the downregulation of MAPK through its inhibitors may represent a potential therapeutic strategy for AD.

## 4. Is the Aβ Hypothesis True?

The aggregation of Aβ in the brain followed by the aggregation of phosphorylated tau protein is the main cause of the disease. But the Aβ hypothesis is questioned by some researchers [100,166]. Espay et al. 2023 [167] suggested that “when protein become aggregated into amyloid state, described as pathology, they not only define neurodegenerative disease, but cause them—pathology is pathogenesis” [167]. Results obtained in a study performed on AD patients with lecanemab, a monoclonal antibody that binds to soluble Aβ protofibrils [168], suggest that this drug “hailed as momentous breakthrough” [169]. On the other hand, according to suggestions of other researchers, the obtained results should be taken with caution: “… lecanemab made a tiny difference to cognitive deterioration on cognition scales over 18 months, which although statistically significant, may not be clinically significant” [170]. The researchers also stated that the mentioned drug revealed moderately less decline on measures of cognition and function, as compared to placebo at 18 months, but unfortunately also revealed adverse effects [168]. Based on suggestions presented by other authors, Espray’s approach is flawed [100,166]. This negative opinion is based on the observation that aggregated proteins are not associated with accurately predicting functional decline. The second argument are the observations that amyloid clearance and the use of lecanemab or donanemab as therapies in AD patients showed a lack of importance, showing only statistically significant slowing in cognitive decline, but at a magnitude below the established values for clinical meaningfulness [100]. There are also several other investigations, which revealed that the concept of the amyloid hypothesis is not valid [166]. For example, several tested drugs decrease amyloid levels in the brain in a dose-dependent manner; no such improvement was found in cognitive tests. It may be concluded that the amyloid is not the main cause of AD development. Therefore, focusing on a single protein that becomes toxic when misfolded due to its aggregation was not a successful strategy for drug development [166].

## 5. Diagnosis Methods of Insulin Resistance and Alzheimer’s Disease

Alzheimer’s disease is associated with insulin resistance. But the diagnoses of IR and AD are different.

### 5.1. Diagnosis Methods of Insulin Resistance

Insulin resistance is related to several metabolic abnormalities, such as obesity, glucose intolerance, type 2 diabetes mellitus, and other metabolic syndromes. To measure blood glucose levels, several methods are used. Primarily, glucose tolerance tests (GTTs), insulin tolerance tests (ITTs), hyperinsulinemic-euglycemic clamp (HEC), continuous infusion of glucose with model assessment (CIGMA), the minimal model technique (MMT), insulin suppression test (IST), and insulin release test [171] are used. GTT shows how quickly exogenous glucose is cleared from the blood [172]. This method is used to diagnose T1DM, T2DM, and gestational diabetes mellitus (GDM). The ITT is used to examine the systemic sensitivity of receptors. In this method, changes in blood glucose levels are measured before and after intravenous insulin administration [173]. This test is suggested to examine of efficacy of compounds and pharmacological agents, which may modify insulin sensitivity. Note that the ITT may often induce hypoglycemia and hypokalaemia. Therefore, these may be systemic counterregulatory responses after intravenous insulin. However, while there are limitations in the cases of the GTT and ITT, these tests are widely used for assessing insulin sensitivity. The HEC test is the gold-standard method to measure insulin sensitivity in vivo [171]. Also, other indices have been developed and test insulin sensitivity/resistance. In clinical research, IR measurements are widely used, such as HOMA2 (updated HOMA model), the Homeostatic Model Assessment for IR (HOMA-IR), the oral glucose insulin sensitivity index (OGSI), fasting insulin (FINS) and fasting plasma glucose (FPG), as well as several others [171].

### 5.2. Diagnosis Methods of Alzheimer’s Disease

Clinically manifested progressive behavioral changes, such as loss of recent memory, declines in executive functions, and defects in cognition, may suggest the development of AD. Higher levels of diagnostic accuracy may be obtained using laboratory tests. There are two main diagnostic methods: neuroimaging, and cerebrospinal fluid and peripheral blood markers.

The clinical and post-mortem investigations may provide the most accurate means of diagnosing AD. For example, magnetic resonance imaging (MRI) of the brain may be used to track the progression of medial temporal lobe atrophy and AD worsening in terms of stage and severity [174]. The progressive cortical hypo-perfusion and hypo-metabolism disturbances detected in the advancement of AD may be detected by single photon emission computed tomography (SPECT) and positron emission tomography (PET) [116,175].

Biomarkers for detecting and diagnosing severity of AD are associated with measurements AβPP-Aβ, tau, and phosphor-tau in cerebrospinal fluid [116,175]. Changes in the levels of Aβ-42, total tau, and phosphor-tau can help predict the progression from mild cognitive impairment (MCI) to dementia, as well as helping in diagnosing AD [116,175]. It is of note that the sensitivity and specificity of investigated biomarkers in CSF approach 85% for the diagnosis of AD and the distinguishing of AD from MCI [116]. In AD, several other significant abnormalities are also observed, such as oxidative stress, neuroinflammation, mitochondrial dysfunction, metabolic disturbances, and an impaired insulin signaling pathway. Investigations of these abnormalities may also be used as biomarkers in the specificity of diagnosing AD. For more information, see [116,175].

## 6. Potential Therapeutic Approach

Several non-pharmacological and pharmacological therapies are used in patients with AD. It has been observed that non-pharmacological therapies may be more effective than pharmacological approaches. However, although several drugs are available for the treatment of AD-dementia, there is still no effective cure for the disease [176].

### 6.1. Non-Pharmacological Therapies

Several non-pharmacological therapies have been suggested. Insulin resistance management is possible through changes in lifestyle, such as dietary changes, increased exercise, and disease prevention [171]. Lifestyle modification (LSM) studies revealed that a diet with physical activity may have a positive role in the therapeutic strategy of managing insulin resistance [177,178]. The proposed lifestyle interventions include physical activity, social care, cognitive stimulation, and dietary interventions. Dietary approaches include calorie-restricted diets, as well as the ketogenic and Mediterranean diets [179]. Nutritional intervention is associated with decreased calories, avoidance of carbohydrates, and focusing on foods with low glycemic index, such as vegetables, fruits, whole-grain products, nuts, lean meats, or beans. Also, higher fiber intake, vitamins, low saturated fat, and proteins are helpful for patients to improve insulin sensitivity. This dietary intervention improves insulin sensitivity and the molecular markers of AD, such as the increase of Aβ42 in the CSF, whose reduced levels are observed in patients with AD. Nutritional intervention has a beneficial effect in slowing the progression of neurodegenerative diseases [178]. Observations have revealed that aerobic fitness, muscular strength, and endurance are improved by aerobic and resistance exercise. The observed changes in muscle function are associated with reduced cognitive decline and brain degeneration caused by decreased gray matter volume. Studies conducted in children and college-aged adults showed that interrupting classroom sitting with physical activity may improve cognitive function and cerebral blood flow. A healthy diet with regular activity in the form of approximately 30 min of exercise at least five days a week activates muscle cells, causing an increase in the activity of AMPK, inactivation of TCB1D1, and the stimulation of GLUT4 translocation to the cell membrane, increasing glucose uptake, which increases insulin reactivity [171]. Increased insulin sensitivity, caused by physical activity, is due to enhanced muscle mitochondrial biogenesis, GLUT4 protein content, and glucose uptake, as well as repartitioning of intramyocellular lipid and reduction in fat mass. For more details, see [177,180]. Animal studies revealed that exercise and physical activity influence brain insulin sensitivity and have a positive effect on insulin signaling in the brain [180]. The effects of these therapeutic strategies are explained by their role in targeting the AD-IR pathogenic link [179]. While LSM remains the key to the prevention and treatment of AD, only a limited number of people continue long-term LSM [177].

### 6.2. Pharmacological Therapies

Unfortunately, there are no treatments that can stop or reverse the progression of AD. Currently used therapies, particularly some medications, may temporarily improve the symptoms of the disease in AD patients [181].

#### 6.2.1. Intranasal Insulin

There are different pathways for the administration of insulin. It may be administered subcutaneously, intramuscularly, or orally. Observations have revealed that in non-diabetic individuals, peripheral administration of insulin may have adverse effects, such as hypoglycemia [182,183]. Peripheral administration of insulin may be ineffective, due to the impaired transport of insulin across the BBB. Intranasal insulin administration (INI) is more effective, as insulin bypasses the BBB. There is the direct neuroanatomical connection between the olfactory nerves and the brain, caused by olfactory nerve channels and trigeminal perivascular channels [181,182]. Observations of the effects of inhaled insulin revealed a beneficial role for this form of therapy in AD patients. Clinical studies have shown that this form of therapy improves cognitive function and memory [181,182,183]. INI improves memory performance in AD patients, particularly in those who are less sensitive to brain insulin signaling [14]. Animal studies revealed that acute insulin delivery to the hippocampus of animal models of AD improves spatial memory by regulating glucose utilization via the PI3K signaling pathway [183]. Unfortunately, there are also other studies that do not confirm these observations [183,184,185]. Therefore, experimental and clinical studies are needed to conclusively establish INI as a therapy for AD patients [4,181,183,185].

#### 6.2.2. Metformin

Metformin is a biguanide antihyperglycemic agent. It is widely used to treat patients with T2DM. It crosses the BBB. Based on its insulin-sensitizing properties, it may improve brain IR and decrease the risk of dementia [183]. Observations have revealed that it may also improve cognitive function. A meta-analysis showed a beneficial effect in diabetic patients with dementia or AD [186]. Animal studies performed on mouse models of AD revealed that metformin improves memory, decreases Aβ concentration and hyperphosphorylated tau, and activates microglia [187,188]. These effects were associated with improved brain insulin signaling [182]. It is suggested that reduced risk of dementia in non-diabetic patients may be due to the influence of metformin on mitochondrial function and the NDUFA2 gene [189]. The discontinuation of metformin in diabetic patients is associated with an increased incidence of dementia [190]. On the other hand, it was observed that in patients with T2DM, long-term use of metformin causes a slightly increased risk of AD [191]. This effect is due to Vitamin B12 deficiency induced by metformin [192].

#### 6.2.3. Glucagon-like Peptide-1 Receptor (GLP-1R) Agonists

GLP-1R agonists, which are insulinotropic hormones, are drugs used in the therapy of patients with T2DM. Liraglutide, a GLP-1R agonist, decreases cognitive impairment. In vitro studies revealed that it regulates neuronal insulin signaling and suppresses the accumulation of Aβ peptide and the phosphorylation of tau protein [193]. Patients with T2DM treated with liraglutide have a reduced risk of dementia compared with those treated with a placebo [194]. Results obtained in animal studies revealed that liraglutide prevents the loss of brain INSR and synapses. It also reverses cognitive impairment caused by Aβ in the mouse hippocampus [195].

#### 6.2.4. Thiazolidinediones

Thiazolidinediones (TZDs) are synthetic peroxisome proliferator-activated receptor-gamma (PPAR-γ). They may improve cognitive function and memory [196]. Rosiglitazone and pioglitazone are the most investigated TZDs as a potential therapy in AD patients. In animal models of AD, rosiglitazone increases IDE levels and decreases Aβ levels [197]. It increases insulin sensitivity and regulates APP processing, leading to decreased plasma Aβ levels [198]. However, there are different and sometimes controversial results regarding the effect of rosiglitazone on cognitive improvement. Pioglitazone increases insulin sensitivity, decreases hippocampal Aβ oligomer, and enhances pro-cognitive effects in animals [199]. Unfortunately, adverse effects of TZDs, such as edema and congestive heart failure, limit their use in dementia and AD [192].

Several other drugs have also been suggested, including sodium-glucose cotransporter-2 inhibitors, phosphodiesterase 5 inhibitors, berberine, guercetin, L-arginine, non-steroidal anti-inflammatory drugs, and antioxidant drugs in the therapy of AD patients. However, these drugs require further clinical and experimental investigation.

## 7. Conclusions

Alzheimer’s disease is a highly prevalent condition affecting people worldwide. A better understanding of the molecular basis of this disease, and especially its molecular processes, will help identify therapeutic targets for AD. Alzheimer’s disease has a multifactorial nature; therefore, it is too diverse to ascribe its origin to a single case. Because insulin resistance is accepted as a pre-AD stage, it cannot be ignored. The treatment strategy of normalizing insulin signaling pathway in the brain may have a potentially key role in the process of stop disease progression. It is also an important strategy to address the underlying mechanisms rather than just focusing on a single disease parameter or on symptom management. The first encouraging results obtained from clinical trials in AD patients revealed that the preclinical results translate into the clinical setting. Perhaps this is a step to reduce or stop several neurodegenerative diseases, including Alzheimer’s disease. The insulin signaling pathway is very important for learning and memory. Unfortunately, in AD, IR disrupts insulin signaling. As a result of impaired insulin signaling, other pathologies are also observed. Brain insulin resistance promotes mitochondrial and cognitive dysfunction, impairing memory. Insulin deficiency and its impaired signaling disrupt brain function. Medications for diabetes mellitus might be repurposed as therapeutics against neuronal degeneration. Anti-diabetic drugs, such as insulin, metformin, thiazolidinediones, gastric inhibitory polypeptide (GIP), and GLP-1 agonists have been investigated for the treatment of neurodegenerative diseases such as AD; however, more robust clinical data are required.

## Data Availability

No new data were created or analyzed in this study. Data sharing is not applicable to this article.

## References

[1] Rahman M.S., Hossain K.S., Das S., Kundu S., Adegoke E.O., Rahman M.A., Hannan M.A., Uddin M.J., Pang M.-G. (2021). Role of insulin in health and disease: An update. Int. J. Mol. Sci..

[2] Saltiel A.R. (2021). Insulin signaling in health and disease. J. Clin. Investig..

[3] Szablewski L. (2024). Insulin resistance: The increased risk of cancers. Curr. Oncol..

[4] Ruderman N.B., Carling D., Prentki M., Cacicedo J.M. (2013). AMPK, insulin resistance, and the metabolic syndrome. J. Clin. Investig..

[5] Sędzikowska A., Szablewski L. (2021). Insulin and insulin resistance in Alzheimer’s disease. Int. J. Mol. Sci..

[6] Craft S., Watson G.S. (2004). Insulin and neurodegenerative disease: Shared and specific mechanisms. Lancet Neurol..

[7] Akhtar A., Sah S.P. (2020). Insulin signaling pathway and related molecules: Role in neurodegeneration and Alzheimer’s disease. Neurochem. Int..

[8] Schulingkamp R., Pagano T., Hung D., Raffa R. (2000). Insulin receptors and insulin action in the brain: Review and clinical implication. Neurosci. Biobehav. Rev..

[9] Ferrannini E., Galvan A.Q., Gastaldelli A., Camastra S., Sironi A.M., Toschi E., Baldi S., Frascerna S., Monzani F., Antonelli A. (1999). Insulin: New roles for an ancient hormone. Eur. J. Clin. Investig..

[10] Heni M. (2024). The insulin resistant brain: Impact on whole-body metabolism and body fat distribution. Diabetologia.

[11] Csajbok E.A., Tamas G. (2016). Cerebral cortex: A target and source of insulin. Diabetologia.

[12] Bromander S., Anckarsäter R., Ahrén B., Kristiansson M., Blennow K., Halmäng A., Zetterberg H., Anckarsäter H., Wass C.E. (2010). Cerebrospinal fluid insulin during non-neurological surgery. J. Neural Transm..

[13] Arnold S.E., Arvanitakis Z., Macauley-Rambach S.L., Koenig A.M., Wang H.-Y., Ahima R.S., Craft S., Gandy S., Buettner C., Stoeckel L.E. (2018). Brain insulin resistance in type 2 diabetes and Alzheimer disease: Concepts and conundrums. Nat. Rev. Neurol..

[14] Hallschmid M. (2021). Intranasal insulin for Alzheimer’s disease. CNS Drugs.

[15] Banks W.A. (2004). The source of cerebral insulin. Eur. J. Pharmacol..

[16] De la Monte S.M. (2013). Intranasal insulin therapy for cognitive impairment and neurodegeneration: Current state of art. Expert Opin. Drug Deliv..

[17] Gerozissis K. (2008). Brain insulin, energy and glucose homeostasis: Genes, environment and metabolic pathologies. Eur. J. Pharmacol..

[18] Benedict C., Hallschmid M., Hatke A., Schulters B., Fehm H.L., Born J., Kern W. (2004). Intranasal insulin improves memory in humans. Psychoneuroendocrinology.

[19] Zhao W., Chen H., Xu H., Moore E., Meiri N., Quon M.J., Alkon D.L. (1999). Brain insulin receptors and spatial memory. Correlated changes in gene expression, tyrosine phosphorylation, and signaling molecules in the hippocampus of water maze trained rats. J. Biol. Chem..

[20] Kopf S.R., Baratti C.M. (1999). Effects of posttraining administration of insulin of a habituation response in mice: Participation of a central cholinergic mechanism. Neurobiol. Learn Mem..

[21] Ponce-Lopez T. (2025). Peripheral inflammation and insulin resistance: Their impact on blood-brain barrier integrity and glia activation in Alzheimer’s disease. Int. J. Mol. Sci..

[22] Kleinridder A., Ferris H.A., Cai W., Kahn C.R. (2014). Insulin action in brain regulates systemic metabolism and brain function. Diabetes.

[23] De Felice F.G., Ferreira S.T. (2014). Inflammation, defective insulin signaling, and mitochondrial dysfunction as common molecular denominators connecting type 2 diabetes to Alzheimer disease. Diabetes.

[24] Tokarz V.L., MacDonald P.E., Klip A. (2018). The cell biology of systemic insulin function. J. Cell. Biol..

[25] Hutton J.C. (1994). Insulin secretory granule biogenesis and the proinsulin-processing endopeptidases. Diabetologia.

[26] Carvalho C., Cardoso S.M., Correira S.C., Moreira P.I. (2019). Tortuous paths of insulin signaling and mitochondria in Alzheimer’s disease. Adv. Exp. Med. Biol..

[27] Neth B.J., Crafts S. (2017). Insulin resistance and Alzheimer’s disease: Bioenergetics linkages. Front. Aging Neurosci..

[28] Bravo D.A., Gleason J.B., Sanchez R.I., Roth R.A., Fuller R.S. (1994). Accurate and efficient cleavage of the human insulin proreceptor by the human proprotein-processing protease furin. Characterization and kinetic parameters using the purified, secreted soluble protease expressed by a recombinant baculovirus. J. Biol. Chem..

[29] Steen E., Terry B.M., Rivera E.J., Cannon J.L., Neely T.R., Tavares R., Xu X.J., Wands J.R., de la Monte S.M. (2005). Impaired insulin and insulin-like growth factor expression and signaling mechanisms in Alzheimer’s disease—Is this type 3 diabetes?. J. Alzheimer’s Dis..

[30] Kido Y., Nakae J., Accili D. (2001). Clinical review 125: The insulin receptor and its cellular targets. J. Clin. Endocrinol. Metab..

[31] Hedo J.A., Kahn C.R., Hayashi M., Yamada K.M., Kasuga M. (1983). Biosynthesis and glycosylation of the insulin receptor. Evidence for a single polypeptide precursor of the two major subunits. J. Biol. Chem..

[32] Choi E., Bai X.-C. (2023). The activation mechanism of the insulin receptor: A structural perspective. Annu. Rev. Biochem..

[33] De Meyts P., Whittaker J. (2002). Structural biology of insulin and IGF1 receptors: Implications for drug design. Nat. Rev. Drug Disc..

[34] De Meyts P., Feingold K.R., Adler R.A., Ahmed S.F., Anawalt B., Blackman M.R., Chrousos G., Corpas E., de Herder W.W., Dhatariya K. (2000). The Insulin Receptor and Its Signal Transduction Network.

[35] Boucher J., Kleinridders A.A., Kahn C.R. (2014). Insulin receptor signaling in normal and insulin-resistant states. Cold Spring Harb. Perspect. Biol..

[36] Mantzoros C., Serdy S. (2023). Insulin action. UpToDate Online Medical Text, Topic 1789.

[37] Leclerc M., Bourassa P., Tremblay C., Cron V., Sugère S., Emond V., Bennett D.A., Calon F. (2023). Cerebrovascular insulin receptors are defective in Alzheimer’s disease. Brain.

[38] Mosthaf L., Grako K., Dull T.J., Coussens L., Ullrich A., McClain D.A. (1990). Functionally distinct insulin receptor generated by tissue-specific alternative splicing. EMBO J..

[39] Milstein J.L., Ferris H.A. (2021). The brain as an insulin-sensitive metabolic organ. Mol. Metab..

[40] Banks W.A., Owen J.B., Erickson M.A. (2012). Insulin in the brain. Pharmacol. Ther..

[41] Ghasenmi R., Haeri A., Dargahi L., Mohamed Z., Ahmadiani A. (2013). Insulin in the brain: Sources, localization and functions. Mol. Neurobiol..

[42] Kochanski R.A. (1993). Insulin receptor autophosphorylation. II. Determination of autophosphorylation by chemical sequence analysis and identification of the juxtamembrane sites. Biochemistry.

[43] White M.F., Shoelson S.E., Keutmann H., Kahn C.R. (1988). A cascade of tyrosine autophosphorylation in the β-subunit activates the phosphotransferase of the insulin receptor. J. Biol. Chem..

[44] Kasuga M., Zick Y., Blithe D.L., Crettaz M., Kahn C.R. (1982). Insulin stimulates tyrosine phosphorylation of the insulin receptor in a cell-free system. Nature.

[45] Ullrich A., Bell J.R., Chen E.Y., Herrera R., Petruzzelli L.M., Dull T.J., Gray A., Coussens L., Liao Y.C., Tsubokawa M. (1985). Human insulin receptor and its relationship to the tyrosine kinase family of oncogenes. Nature.

[46] Kahn C.R., White M.F. (1988). The insulin receptor and the molecular mechanism of insulin action. J. Clin. Investig..

[47] Sun X.J., Rothenberg P., Kahn C.R., Backer J.M., Araki E., Wilden P.A., Cahill D.A., Goldstein B.J., White M.F. (1991). Structure of the insulin receptor substrate IRS-1 defines a unique signal transduction protein. Nature.

[48] White M.F., Maron R., Kahn C.R. (1985). Insulin rapidly stimulates tyrosine phosphorylation of a *M*_r_-185,000 protein in intact cells. Nature.

[49] Lavan B.E., Lane W.S., Lienhard G.E. (1997). The 60-kDa phosphotyrosine protein in insulin-treated adipocytes is a new member of the insulin receptor substrate family. J. Biol. Chem..

[50] Sun X.J., Wang L.M., Zhang Y., Yenush L., Myers M.G., Glasheen E., Lane W.S., Pierce J.H., White M.F. (1995). Role of IRS-2 in insulin and cytokine signaling. Nature.

[51] White M.F. (2003). Insulin signaling in health and disease. Science.

[52] Cai D., Dhe-Paganon S., Melendez P.A., Lee J., Shoelson S.E. (2003). Two new substrates in insulin signaling, IRS/DOK4 and IRS6/DOK. J. Biol. Chem..

[53] Bjornholm M., He A.R., Attersand A., Lake S., Liu S.C.H., Lienhard G.E., Taylor S., Anner P., Zierath J.R. (2002). Absence of functional insulin receptor substrate-3 (IRS-3) gene in humans. Diabetologia.

[54] Myers M.G., White M.F. (1995). New frontiers in insulin receptor substrate signaling. Trends Endocrinol. Metab..

[55] Taniguchi C.M., Emanuelli B., Kahn C.R. (2006). Critical nodes in signaling pathways: Insights into insulin action. Nat. Rev. Mol. Cell Biol..

[56] Vadas O., Burke J.E., Zhang X., Berndt A., Williams R.L. (2011). Structural basis for activation and inhibition of class I phosphoinositide 3-kinases. Sci. Signal..

[57] Shaw L.M. (2011). The insulin receptor substrate (IRS) proteins: At the intersection of metabolism and cancer. Cell Cycle..

[58] Gallay N., Dos Santos C., Cuzin L., Bousquet M., Gouy V.S., Chaussade C., Attal M., Payrastre B., Demur C., Recher C. (2009). The level of AKT phosphorylation on threonine 308 but not on serine 473 is associated with high-risk cytogenetics and predicts poor overall survival in acute myeloid leukemia. Leukemia.

[59] Han E.K., Leverson J., McGonigal T., Shah O., Woods K., Hunter T., Giranda V., Luo Y. (2007). Akt inhibitor A-443654 induces rapid Akt Ser-473 phosphorylation independent of mTOR inhibition. Oncogene.

[60] Paez J., Sellers W.R. (2004). PI3K/PTEN/Akt Pathway, Signal Transduction in Cancer.

[61] Easton R.M., Cho H., Roovers K., Shineman D.W., Mizrahi M., Forman M.S., Lee V.M.-Y., Szaboles M., de Jong R., Oltersdorf T. (2005). Role of Akt3/protein kinase Bγ in attainment of normal brain size. Mol. Cell. Biol..

[62] Tang Y., Huang B., Sun L., Peng X., Chen X., Zou X. (2011). Ginkgolide B promotes proliferation and functional activities of bone marrow-derived endothelial progenitor cells: Involvement of Akt/eNOS and MAPK/p38 signaling pathways. Eur. Cells Mater..

[63] Saito Y., Tanaka Y., Aita Y., Ishii K.-A., Ikeda K., Kawakami Y., Shimano H., Hara H., Takekoshi K. (2011). Sunitinib induces apoptosis in pheochromocytoma tumor cells by inhibiting VEGFR2/Akt/mTOR/S6K1 pathways through modulation of Bcl-2 and BAD. Am. J. Physiol. Endocrinol. Metab..

[64] Harris T.E., Lawrence J.C. (2003). TOR signaling. Sci. STKE.

[65] Nguyen P.V. (2002). Protein synthesis during LTP: Linking synaptic activity to translation. Trends Neurosci..

[66] Tang S.J., Reis G., Kang H., Gingras A.C., Sonenberg N., Schuman E.M. (2002). A rapamycin-sensitive signaling pathway contributes to long-term synaptic plasticity in the hippocampus. Proc. Natl. Acad. Sci. USA.

[67] Huang H.C., Tang D., Xu K., Jiang Z.F. (2014). Curcumin attenuates amyloid-β-induced tau hyperphosphorylation in human neuroblastoma SH-SY5Y cells involving PTEN/Akt/GSK-3β signaling pathway. J. Recept. Signal Transduct. Res..

[68] Min H.J., Koh S.S., Cho I.R., Srisuttee R., Park E.H., Jhun B.H., Kim Y.G., Oh S., Kwak J.E., Johnston R.N. (2009). Inhibition of GSK-3β enhances reovirus-induced apoptosis in colon cancer cells. Int. J. Oncol..

[69] Ding V.W., Chen R.H., McCormick F. (2000). Differential regulation of glycogen synthase kinase 3β by insulin and Wnt signaling. J. Biol. Chem..

[70] Svendsen A.M., Winge S.B., Zimmermann M., Lindvig A.B., Warzecha C.B., Sajid W., Horne M.C., De Meyts P. (2014). Downregulation of cyclin G2 by insulin, IGF-1 (insulin-like growth factor 1) and X10 (Asp B10 insulin): Role in mitogenesis. Biochem. J..

[71] Sano H., Kane S., Sano E., Miinea C.P., Asara J.M., Lane W.S., Garner C.W., Lienhard G.E. (2003). Insulin-stimulated phosphorylation of a Rab GTPase-activiting protein regulates GLUT4 translocation. J. Biol. Chem..

[72] Taylor E.B., An D., Kramer H.F., Yu H., Fujii N.L., Roeckel K.S., Bowles N., Hirshman M.F., Xie J., Feener E.P. (2008). Discovery of TBC1D1 as an insulin-, AICR-, and contraction-stimulated signaling nexus in mouse skeletal muscle. J. Biol. Chem..

[73] An D., Toyoda T., Taylor E.B., Yu H., Fujii N., Hirshman M.E., Goodyear L.J. (2010). TBC1D1 regulates insulin- and contraction-induced glucose transport in mouse skeletal muscle. Diabetes.

[74] Skolnik E.Y., Lee C.H., Batzer A. (1993). The SH2/SH3 domain-containing protein GRB2 interacts with tyrosine-phosphorylated IRS1 and Shc: Implications for insulin control of Ras signaling. EMBO J..

[75] Gureasko A., Galush W.J., Boykevisch S., Sondermann H., Bar-Sagi D., Groves J.T., Kurlyan J. (2008). Membrane-dependent signal integration by the Ras activator or Son of sevenless. Nat. Struct. Mol. Biol..

[76] Aviello G., Rowland L., Gill C.I., Acquaviva A.M., Capasso F., McCann M., Capasso R., Izzo A.A., Borrelli F. (2010). Anti-proliferative effect of rhein, an antraquinone isolated from Cassia species, on Caco-2 human adenocarcinoma cells. J. Cell. Mol. Med..

[77] Haeusler R.A., McGraw T.E., Accili D. (2018). Biochemical and cellular properties of insulin receptor signaling. Nat. Rev. Mol. Cell Biol..

[78] Racaniello M., Cardinale A., Mollinari C., D’Antuono M., De Chiara G., Tancredi V., Merlo D. (2010). Phosphorylation changes of CaMKII, ERK1/2, PKB/Akt kinase and CREB activation during long-term potentiation of Schaffer collateral-CA1 mouse hippocampal synapses. Neurochem. Res..

[79] Hu Y., Russek S.J. (2008). BDNF and the diagnosed nervous system: A delicate balance between adaptation and pathological process of gene regulation. J. Neurochem..

[80] Di Camillo B., Carlon A., Eduati F., Toffolo G.M. (2016). A rule-based model of insulin signaling pathway. BMC Syst. Biol..

[81] Kolb H., Kempf K., Röhling M., Martin S. (2020). Insulin: Too much of a good think is bad. BMC Med..

[82] Kim A.B., Arvanitakis Z. (2023). Insulin resistance, cognition, and Alzheimer disease. Obesity.

[83] Andrade L.J.O., Oliveira L.M., Bittencourt A.M.V., Lourenço L.G.C., Oliveira G.C.M. (2024). Brain insulin resistance and Alzheimer’s disease: A systematic review. Dev. Neuropsychol..

[84] Sun Y., Ma C., Sun H., Wang H., Peng W., Zhou Z., Wang H., Pi C., Shi Y., He H. (2020). Metabolism: A novel shared link between diabetes mellitus and Alzheimer’s disease. J. Diabetes Res..

[85] Du X., Wang X., Geng M. (2018). Alzheimer’s disease, hypothesis and related therapies. Transl. Neurodegener..

[86] Hebert L.E., Scherr P.A., Bienias J.L., Bennett D.A., Evans D.A. (2003). Alzheimer disease in the USA population: Prevalence estimates using the 2000 census. Arch. Neurol..

[87] Hebert L.E., Weuve J., Scherr P.A., Evans D.A. (2013). Alzheimer diseases in the United States (2010–2050) estimated using the 10 census. Neurology.

[88] Alzheimer’s Association (2016). 2016 Alzheimer’s disease facts and figures. Alzheimer’s Dement..

[89] Holtzman D.M., Morris J.C., Goate A.M. (2011). Alzheimer’s disease: The challenge of the second century. Sci. Transl. Med..

[90] Selkoe D.J. (1996). Amyloid β-protein and the genetics of Alzheimer’s disease. J. Biol. Chem..

[91] Roher A.E., Maarouf C.L., Kokjohn T.A. (2016). Familial presenilin mutations and sporadic Alzheimer’s disease pathology: Is the assumption of biochemical equivalence justified?. J. Alzheimer’s Dis..

[92] Chen Y., Strickland M.R., Sorrano A., Holtzman D.M. (2021). Apolipoprotein E: Structural insights and links to Alzheimer disease pathogenesis. Neuron.

[93] Rhea E.M., Raber J., Banks W.A. (2020). ApoE and cerebral insulin: Trafficking, receptors and resistance. Neurobiol. Dis..

[94] Patel V.N., Chorawala M.R., Shah M.B., Shah K.C., Dave B.P., Shah M.P., Patel T.M. (2022). Emerging pathophysiological mechanisms linking diabetes mellitus and Alzheimer’s disease: An old wine in a new bottle. J. Alzheimer’s Dis. Rep..

[95] Irie F., Fitzpatrick A.L., Lopez O.L., Kuller L.H., Peila R., Newman A.B., Launer L.J. (2008). Enhanced risk for Alzheimer disease in person with type 2 diabetes and APOE. Arch. Neurol..

[96] Sima A.A.A., Li Z. (2006). Diabetes and Alzheimer’s disease—Is there a connection. Rev. Diabet. Stud..

[97] Karch C.M., Jeng A.T., Nowotny P., Cady J., Cruchaga C., Goate A.M. (2012). Expression of novel Alzheimer’s disease risk genes in control and Alzheimer’s disease brains. PLoS ONE..

[98] Alzheimer’s Association (2015). 2015 Alzheimer’s disease facts and figures. Alzheimer’s Dement..

[99] Stanley M., Macauley S.L., Holtzman D.M. (2016). Changes in insulin and insulin signaling in Alzheimer’s disease: Cause or consequence?. J. Exp. Med..

[100] Winter G. (2025). Insulin resistance may hold the key to Alzheimer’s disease. Br. J. Nurs..

[101] Akar E., Kaçar M. (2023). Alzheimer’s disease and insulin relationship: Type 3 diabetes. Haydarpaşa Numune Med. J..

[102] Dubois B., Hampel H., Feldman H.H., Schelters P., Alsen P., Andrieu S., Bakardjian H., Benali H., Bertram L., Blennow K. (2016). The Preclinical Study of July, U.S.A. Washington De, Preclinical Alzheimer’s disease: Definition, natural history, and diagnostic criteria. Alzheimer’s Dement..

[103] Serrano-Pozo A., Frosch M.P., Masliah E., Hyman B.T. (2011). Neuropathological alterations in Alzheimer’s disease. Cold Spring Harb. Perspect. Med..

[104] Abdella M.M.I. (2024). Insulin resistance as the molecular link between diabetes and Alzheimer’s disease. World J. Diabetes.

[105] Rumble B., Retallack R., Hilbich C., Simms G., Multhaup G., Martins R., Hockey A., Montgomery P., Beyreuther K., Masters C.L. (1989). Amyloid A4 protein and its precursor in Down’s syndrome and Alzheimer’s disease. N. Engl. J. Med..

[106] Glenner G.G., Wong C.W. (1984). Alzheimer’s disease and Down’s syndrome: Sharing of a unique cerebrovascular amyloid fibril protein. Biochem. Biophys. Res. Commun..

[107] Zhang Y.W., Thompson R., Zhang H., Hu H. (2011). APP processing in Alzheimer’s disease. Mol. Brain..

[108] Head E., Powell D., Gold B.T., Schmitt F.A. (2012). Alzheimer’s disease in Down syndrome. Eur. J. Neurodegener. Dis..

[109] Hardy J., Selkoe D.J. (2002). The amyloid hypothesis of Alzheimer’s disease: Progress and problems on the road to therapeutic. Science.

[110] Alves S.S., da Silva-Junior R.M.P., Servilha-Menezes G., Homolak J., Šalković-Petrišič M., Garcia-Cairasco N. (2021). Insulin resistance as a common link between current Alzheimer’s disease hypotheses. J. Alzheimer’s Dis..

[111] Tan J.Z.A., Gleeson P.A. (2019). The role of membrane precursor protein and production of amyloid peptides in Alzheimer’s disease. Biochim. Biophys. Acta Biomembr..

[112] Pandini G., Pace V., Copani A., Squatrito S., Vigneri R. (2013). Insulin has multiple antiamyloidogenic effects on human neuronal cells. Endocrinology.

[113] Burillo J., Marqués P., Jiménez B., González-Blanco C., Benito M., Guillén C. (2021). Insulin resistance and diabetes mellitus in Alzheimer’s disease. Cells.

[114] Rad S.K., Arya A., Karimian H., Madhavan P., Rizwan F., Koshy S., Prabhu G. (2018). Mechanism involved in insulin resistance via accumulation of β-amyloid and neurofibrillary tangles: Link between type 2 diabetes and Alzheimer’s disease. Drug Des. Dev. Ther..

[115] Walsh D.M., Klyubin I., Fadeeva J.V., Cullen W.K., Anwyl R., Wolfe M.S., Rowan M.J., Selkoe D.J. (2002). Naturally secreted oligomers of amyloid β protein potently inhibit hippocampal long-term potentiation in vivo. Nature.

[116] de la Monte S. (2012). Contributions of brain insulin resistance and deficiency in amyloid-related neurodegeneration in Alzheimer’s disease. Drugs.

[117] Hoe H.S., Lee H.K., Pak D.T. (2012). The upside of APP at synapses. CNS Neurosci. Ther..

[118] Berlanga-Acosta J., Guillén-Nieto G., Rodriguez-Rodriguez N., Bringas-Vegá M.L., Garcia-del-Barcó-Herrera D., Berlanga-Saez J.O., Garcia-Ojólvo A., Valdés-Sosa M.J., Valdés-Sosa P.A. (2020). Insulin resistance at the crossroad of Alzheimer’s disease pathology: A review. Front. Endocrinol..

[119] Klyubin I., Cullen W.K., Hu N.W., Rowan M.J. (2012). Alzheimer’s disease Aβ assemblies of synaptic mediating rapid disruption of synaptic plasticity and memory. Mol. Brain.

[120] Rajendran L., Paolicelli R.C. (2018). Microglia-mediated synapse loss in Alzheimer’s disease. J. Neurosci..

[121] Solke D.J., Hardy J. (2016). The amyloid hypothesis of Alzheimer’s disease at 25 years. EMBO Mol. Med..

[122] Forny-Germano L., Lyra e Silva N.M., Batista A.F., Brito-Moreira J., Gralle M., Boehnke S.E., Coe B.C., Lablans A., Marques S.A., Martinez A.M. (2014). Alzheimer’s like pathology induced by amyloid-ε oligomers in nonhuman primates. J. Neurosci..

[123] Dahiya M., Yadov M., Goyal C., Kumar A. (2025). Insulin resistance in Alzheimer’s disease: Signaling mechanisms and therapeutic strategies. Immunopharmacology.

[124] Farris W., Mansourian S., Chang Y., Lindsley L., Eckman E.A., Frosch M.P., Eckman C.B., Tanzi R.E., Selkoe D.J., Guenette S. (2003). Insulin-degrading enzyme regulates the levels of insulin, amyloid β-protein, and the beta-amyloid precursor protein intracellular domain in vivo. Proc. Natl. Acad. Sci. USA.

[125] Lynch J.A., George A.M., Eisenhauer P.B., Conn K., Gao W., Carreras I., Wells I.M., McKee A., Ullman M.D., Fine R.E. (2006). Insulin degrading enzyme is localized predominantly at the cell surface and unpolarized human cerebrovascular endothelial cell cultures. J. Neurosci. Res..

[126] Zhao L., Teter B., Morihara T., Lim G.P., Ambegaokar S.S., Ubeda O.J., Frautschy S.A., Cole G.M. (2004). Insulin-degrading enzyme as a downstream target of insulin receptor signaling cascade: Implications for Alzheimer’s disease intervention. J. Neurosci..

[127] Rensink A.A., Otte-Holler I., de Boer R., Bosch R.R., ten Donkelaar H.J., de Waal R.M., Verbeek M.M., Kremer B. (2004). Insulin inhibits amyloid beta-induced cell death in cultured human brain pericytes. Neurobiol. Aging..

[128] Lee H.K., Kumar P., Fu Q., Rosen K.M., Querfurth H.W. (2009). The insulin/Akt signaling pathway is targeted by intracellular beta-amyloid. Am. Biol. Cell.

[129] Talbot K., Wang H.Y., Kazi H., Han L.-Y., Bakshi K.P., Stucky A., Fuino R.L., Kawaguchi K.R., Samoyedny A.J., Wilson R.S. (2012). Demonstrated brain insulin resistance in Alzheimer’s disease patients, is associated with IGF-1 resistance, IRS-1 dysregulation, and cognitive decline. J. Clin. Investig..

[130] Pritchard S.M., Dolan P.J., Vitkus A., Johnson G.V. (2011). The toxicity of Tau in Alzheimer disease: Turnover, targets and potential therapeutics. J. Cell. Mol. Med..

[131] Goedert M., Spillantini M.G. (2006). A century of Alzheimer’s disease. Science.

[132] Cassimeris L., Spittle C. (2001). Regulation of microtubule-associated proteins. Int. Rev. Cytol..

[133] Arendt T., Stieler J.T., Holzer M. (2016). Tau and tauopathies. Brain Res. Bull..

[134] Pooler A.M., Phillips E.C., Lau D.H., Noble W., Hanger D.P. (2013). Physiological release of endogenous tau is stimulated by neuronal activity. EMBO Rep..

[135] Hong X.P., Peng C.X., Wei W., Tian Q., Liu Y.H., Yao X.Q., Zhang Y., Cao F.Y., Wang Q., Wang J.Z. (2010). Essential role of tau phosphorylation in adult hippocampal neurogenesis. Hippocampus.

[136] Pallas-Bazara N., Jurado-Arjona J., Navarrete M., Esteban J.A., Hernández F., Ávila J., Llorens-Martin M. (2016). Novel function of tau in regulating the effects of external stimuli on adult hippocampal neurogenesis. EMBO J..

[137] Dawson H.N., Ferreira A., Eyster M.V., Ghoshal N., Binder L.I., Vitek M.P. (2001). Inhibition of neuronal maturation in primary hippocampal neurons from tau deficient mice. J. Cell Sci..

[138] Sapir T., Frotscher M., Levy T., Mandelkow E.M., Reiner O. (2012). Tau’s role in the developing brain: Implications for intellectual disability. Hum. Mol. Genet..

[139] Yi S., Liu Q., Wang X., Qiang T., Wang H., Zha G., Yu J., Wang P., Gu X., Chu D. (2019). Tau modulates Schwann cell proliferation, migration and differentiation following peripheral nerve injury. J. Cell Sci..

[140] Kimura T., Whitcomb D.J., Jo J., Regan P., Piers T., Heo S., Brown C., Hashikawa T., Murayama M., Seok H. (2014). Microtubule-associated protein tau is essential for long-term depression in hippocampus. Philos. Trans. R. Soc. B Biol. Sci..

[141] Kent S.A., Spires-Jonses T.L., Durrant C.S. (2020). The physiological roles of tau and Aβ: Implications for Alzheimer’s disease pathology and therapeutics. Acta Neuropathol..

[142] Biundo F., Del Prete D., Zhang H., Arancio O., D’Adamio L. (2018). A role for tau in learning, memory and synaptic plasticity. Sci. Rep..

[143] Lei P., Ayton S., Finkelstein D.I., Spoerri L., Ciccotosto G.D., Wright D.K., Wong B.X.W., Adlard P.A., Cherny R.A., Lam L.Q. (2012). Tau deficiency induces parkinsonism with dementia by impairing APP-mediated iron export. Nat. Med..

[144] Grundke-Iqbal I., Iqbal K., Tung Y.C. (1986). Abnormal phosphorylation of the microtubule-associated protein τ (tau) in Alzheimer cytoskeletal pathology. Proc. Natl. Acad. Sci. USA.

[145] Iqbal K., Alonso Adel C., Chen S., Chohan M.O., El-Akkad E., Gong C.X., Khatoon S., Li B., Liu F., Rahman A. (2005). Tau pathology in Alzheimer disease and other tauopathies. Biochim. Biophys. Acta.

[146] Drewes G., Mandelkow E.M., Baumann K., Goris J., Merlevede W., Mandelkow E. (1993). Dephosphorylation of tau protein and Alzheimer paired helical filaments by calcineurin and phosphatase-2A. FEBS Lett..

[147] Liu F., Grundke-Iqbal I., Iqbal K., Gong C.X. (2005). Contributions of protein phosphatases PP1, PP2A, PP2B and PP5 to the regulation of tau phosphorylation. Eur. J. Neurosci..

[148] Marciniak E., Leboucher A., Caron E., Ahmed T., Tailleux A., Dumont J., Issad T., Gerhardt E., Pagesy P., Vilano M. (2017). Tau deletion promotes brain insulin resistance. J. Exp. Med..

[149] Rodriguez-Rodriguez A., Sandebring-Matton A., Merino-Serrais P., Parrado-Fernandez C., Rabano A., Winblad B., Ávila J., Ferrer I., Cedazo-Minguez A. (2017). Tau hyperphosphorylation induces oligomeric insulin accumulation and insulin resistance in neurons. Brain.

[150] Bertacca A., Ciccarone A., Cechetti P., Vianello B., Laurenza I., Maffei M., Chiellini C., Del Prato S., Benzi L. (2005). Continually high insulin levels impair Akt phosphorylation and glucose transport in human myoblasts. Metabolism.

[151] Catalano K.J., Maddux B.A., Szary J., Youngreen J.F., Goldfine I.D., Schaufele F. (2014). Insulin resistance induced by hyperinsulinemia coincides with a persistent alteration at the insulin receptor tyrosine kinase domain. PLoS ONE.

[152] Liu Y., Liu F., Grundke-Iqbal I., Iqbal K., Gong C.X. (2011). Deficient brain insulin signaling pathway in Alzheimer’s disease and diabetes. J. Pathol..

[153] Moloney A.M., Griffin R.J., Timmons S., O’Connor R., Ravid R., O’Neill C. (2010). Defects in IGF-1 receptor and IRS-1/2 in Alzheimer’s disease indicate possible resistance to IGF-1 and insulin signaling. Neurobiol. Aging.

[154] Serenó L., Coma M., Rodríguez M., Sánchez-Ferrer P., Sánchez M.B., Gich I., Agulló J.M., Pérez M., Avila J., Guardia-Laguarta C. (2009). A novel GSK-3beta inhibitor reduces Alzheimer’s pathology and rescues neuronal loss in vivo. Neurobiol. Dis..

[155] Barone E., Di Domenico F., Perluigi M., Butterfield D.A. (2021). The interplay among oxidative stress, brain insulin resistance and AMPK dysfunction contribute to neurodegeneration in type 2 diabetes and Alzheimer disease. Free Rad. Biol. Med..

[156] Liu Y.J., Chern Y. (2015). AMPK-mediated regulation of neuronal metabolism and function in brain diseases. J. Neurogenet..

[157] Cai Z., Yan L.J., Li K., Quazi S.H., Zhao B. (2012). Roles of AMP-activated protein kinase in Alzheimer’s disease. Neuromol. Med..

[158] Seixas da Silva G.S., Melo H.M., Lourenco M.V., Lyra E.S.N.M., de Carvalho M.B., Alves-Leon S.V., de Souza J.M., Klein W.L., da-Silva W.S., Ferreira S.T. (2017). Amyloid–beta oligomers transiently inhibit AMP-activated kinase and cause metabolic defects in hippocampal neurons. J. Biol. Chem..

[159] Anderson N.J., King M.R., Delbruck L., Jolivalt C.G. (2014). Role of insulin signaling impairment, adiponectin and dyslipidemia in peripheral and central neuropathy in mice. Dis. Model Mech..

[160] Cheng K.K., Lam K.S., Wang B., Xu A. (2014). Signaling mechanisms underlying the insulin-sensitizing effects of adiponectin. Best Pract. Res. Clin. Endocrinol. Metab..

[161] Chen M., Huang N., Liu J., Huang J., Shi J., Jin F. (2021). AMPK: A bridge between diabetes mellitus and Alzheimer’s disease. Behav. Brain Res..

[162] Yang L., Jiang Y., Shi L., Zhong D., Li Y., Li J., Jin R. (2020). AMPK: Potential therapeutic target for Alzheimer’s disease. Curr. Protein Pept. Sci..

[163] Vingtdeux V., Davies P., Dickson D.W., Marambaud P. (2011). AMPK is abnormally activated in tangle- and pre-tangle-bearing neurons in Alzheimer’s disease and other tauopathies. Acta Neuropathol..

[164] Wang X., Zimmermann H.R., Ma T. (2019). Therapeutic potential of AMP-activated protein kinase in Alzheimer’s disease. J. Alzheimer’s Dis..

[165] Munoz L., Ammit A.J. (2010). Targeting p38 MAPK pathway for the treatment of Alzheimer’s disease. Neuropharmacology.

[166] Hölscher C. (2020). Brain insulin resistance: Role of neurodegenerative disease and potential for targeting. Expert Opin. Investig. Drugs.

[167] Espay A.J., Herrup K., Keep K.P., Daly T. (2023). The proteopenia hypothesis: Loss of Aβ42 and the onset of Alzheimer’s disease. Ageing Res. Rev..

[168] van Dyck C.H., Swanson C.J., Aisen P., Bateman R.J., Chen C., Gee M., Kanekiyo M., Li D., Reyderman R.J., Chen C. (2022). Lecanemab in early Alzheimer’s disease. N. Engl. J. Med..

[169] Gallagher J. (2022). Alzheimer’s Drug Lecanemab Hailed as Momentous Breakthrough. BBC News.

[170] Kmietowicz Z. (2022). Why press releases don’t tell the whole story. Br. Med. J..

[171] Li M., Chi X., Wang Y., Setrerrahmane S., Xie W., Hu H. (2022). Trends in insulin resistance: Insights into mechanisms and therapeutic strategy. Signal Transduct. Target. Ther..

[172] Stumvoll M., Mitrakou A., Pimenta W., Jenssen T., Yki-Jarvinen H., van Haeften T.W., Renn W., Gerich J. (2000). Use of the oral glucose tolerance to assess insulin release and insulin sensitivity. Diabetes Care.

[173] Zhang Y., Sun S., Jia H., Qi Y., Zhang J., Lin L., Chen Y., Wang W., Ning G. (2020). The optimized calculation method for insulin dosage in an Insulin Tolerance Test (ITT): A randomized parallel control study. Front. Endocrinol..

[174] Meyer J.S., Huang J., Chowdhury M. (2005). MRI abnormalities associated with mild cognitive impairments of vascular (VMCI) versus neurodegenerative (NMCI) types prodromal for vascular and Alzheimer’s dementias. Curr. Alzheimer Dis..

[175] de la Monte S. (2017). Insulin resistance and neurodegeneration: Progress towards the development of new therapeutics for Alzheimer’s disease. Drugs.

[176] Gutierrez-Tordera M.S., Panisello L., Garcia-Gonzalez B.S., Ruiz A., Cantero J.L., Rojas-Criollo M.S., Mursil M., Atenza M., Novau-Ferre N., Mateu-Fabregat M.S. (2025). Metabolic signature of insulin resistance and risk of Alzheimer’s disease. J. Gerontol. Ser. A Biol. Sci. Med. Sci..

[177] Mastrolotaro L., Roden M. (2021). Insulin resistance and insulin sensitizing agents. Metab. Clin. Exp..

[178] Rojas M., Chávez-Castillo M., Bautista J., Ortega Á., Nava M., Salazar J., Diez-Camargo E., Medina O., Rojas-Quintero J., Bermúdez V. (2021). Alzheimer’s disease and type 2 diabetes mellitus: Pathophysiologic and pharmacotherapeutics links. World J. Diabetes.

[179] Amin A.M., Mostafa H., Khojah H.M.J. (2023). Insulin resistance in Alzheimer’s disease: The genetics and metabolomics links. Clin. Chim. Acta.

[180] Malin S.K., Stewart N.R., Ude A.A., Alderman B.L. (2022). Brain insulin resistance and cognitive function: Influence of exercise. J. Appl. Physiol..

[181] Wei Z., Koya J., Reznik S.E. (2021). Insulin resistance exacerbates Alzheimer disease via multiple mechanisms. Front. Neurosci..

[182] Kellar D., Craft S. (2020). Brain insulin resistance in Alzheimer’s disease and related disorders: Mechanisms and therapeutic approaches. Lancet Neurol..

[183] Affuso F., Micillo F., Fazio S. (2024). Insulin resistance, a risk factor for Alzheimer’s disease: Pathological mechanism and a new proposal for a preventive therapeutic approach. Biomedicines.

[184] Craft S., Raman R., Chow T.W., Rafii M.S., Sun C.K., Rissman R.A., Donohue M.C., Brewer J.B., Jenkins C., Harless K. (2020). Safety, efficacy, and feasibility of intranasal insulin for the treatment of mild cognitive impairment in Alzheimer disease dementia: A randomized clinical trial. JAMA Neurol..

[185] Andrade L.J.O., Matos G., Matos de Oliveira L. (2025). Intranasal insulin in Alzheimer disease (diabetes in situ?): A systematic review and meta-analysis. Dement. Neuropsychol..

[186] Lin Y., Wang K., Ma C., Wang X., Gong Z., Zhang R., Zang D., Cheng Y. (2018). Evaluation of metformin on cognitive improvement in patients with non-dementia vascular cognitive impairment and abnormal glucose metabolism. Front. Aging Neurosci..

[187] Ow Z., Kong X., Sun X., He X., Zhang L., Gong Z., Huang J., Xu B., Long D., Li J. (2018). Metformin treatment prevents amyloid plaque deposition and memory impairment in APP/PS1 mice. Brain Behav. Immun..

[188] Farr S.A., Koesler E., Niehoff M.L., Roby I.A., McKee A., Morley J.E. (2018). Metformin improves learning in the SAMP8 mouse model of Alzheimer’s disease. J. Alzheimer’s Dis..

[189] Zheng J., Xu M., Walker V., Yuan J., Korologou-Linden R., Robinson J., Huang P., Burges S., Au Yeung S.L., Luo S. (2022). Evaluating the efficacy and mechanism of metformin on reducing Alzheimer’s disease risk in the general population: A Mendelian randomized study. Diabetologia.

[190] Zimmerman S.C., Ferguson E.L., Choudhary V., Ranatunga D.K., On-Orison A., Hayes-Larson E., Folle A.D., Mayeda E.R., Whitmer R.A., Gilsanz P. (2023). Metformin cessation and dementia incidence. JAMA Netw. Open.

[191] Imfeld P., Bodmer M., Jick S.S., Meier C.R. (2012). Metformin, other antidiabetic drugs, and risk of Alzheimer’s disease: A population-based case control study. J. Am. Geriatr..

[192] Campbell J.M., Stephenson M.D., de Courten B., Chapman I., Bellman S.M., Aromataris E. (2018). Metformin use associated with reduced risk of dementia in patients with diabetes: A systematic review and meta-analysis. J. Alzheimer’s Dis..

[193] Jantrapirom S., Nimlamool W., Chattipakorn N., Chattipakorn S., Temviriyanurkul P., Inthachat W., Govitrapong P., Potikanond S. (2020). Liraglutide suppresses tau hyperphosphorylation, amyloid beta accumulation through neuronal insulin signaling and BACE-1 activity. Int. J. Mol. Sci..

[194] Nørgaard C.H., Frierich S., Hansen C.T., Gerds T., Ballard C., Møller D.V., Knudsen L.B., Kvist K., Zinman B., Holm E. (2022). Treatment with glucagon-like peptide-1 receptor agonists and incidence of dementia: Data from pooled double-blind randomized controlled trials and nationwide disease and prescription register. Alzheimer’s Dement..

[195] Batista A.F., Forny-Germano L., Clarke J.R., Lyra E Silva N.M., Brito-Moreira J., Boehnke S.E., Winterborn A., Coe B.C., Lablans A., Vital J.F. (2018). The diabetes drug liraglutide reverses cognitive impairment in mice and alternates insulin receptor and synaptic pathology in a non-human primate model of Alzheimer’s disease. J. Pathol..

[196] Khan M.A., Alam Q., Haque A., Ashafaq A., Khan M.J., Ashraf G.M., Ahmad M. (2019). Current progress on peroxisome proliferator-activated receptor gamma agonist as an emerging therapeutic approach for the treatment of Alzheimer’s disease: An update. Curr. Neuropharmacol..

[197] Li H., Wu J., Zhu L., Sha L., Yang S., Wei J., Ji L., Tang W., Mao M., Cao L. (2018). Insulin degrading enzyme contributes to the pathology of type 2 diabetes and Alzheimer’s disease: Possible mechanisms of IDE in T2D and AD. Biosci. Rep..

[198] Pardeshi R., Bolshette N., Godhave K., Ahire A., Ahmed S., Cassano T., Gupta V.B., Lahkar M. (2017). Insulin signaling: An opportunistic target to minify risk of Alzheimer’s disease. Psychoneuroendocrinology.

[199] Gad E.S., Zaitone S.A., Moustafa Y.M. (2015). Pioglitazone and downregulate hippocampal beta amyloid oligomer and microglia expression in insulin-resistant rats. Can. J. Physiol. Pharmacol..

